# Deep Learning in MRI-guided Radiation Therapy: A Systematic Review

**Published:** 2023-03-30

**Authors:** Zach Eidex, Yifu Ding, Jing Wang, Elham Abouei, Richard L.J. Qiu, Tian Liu, Tonghe Wang, Xiaofeng Yang

**Affiliations:** 1 Department of Radiation Oncology and Winship Cancer Institute, Emory University, Atlanta, GA; 2 School of Mechanical Engineering, Georgia Institute of Technology, Atlanta, GA; 3 Department of Radiation Oncology, Icahn School of Medicine at Mount Sinai, New York, NY; 4 Department of Medical Physics, Memorial Sloan Kettering Cancer Center, New York, NY

**Keywords:** MRI-guided, Radiation Therapy, Radiotherapy, Deep Learning, Review

## Abstract

MRI-guided radiation therapy (MRgRT) offers a precise and adaptive approach to treatment planning. Deep learning applications which augment the capabilities of MRgRT are systematically reviewed. MRI-guided radiation therapy offers a precise, adaptive approach to treatment planning. Deep learning applications which augment the capabilities of MRgRT are systematically reviewed with emphasis placed on underlying methods. Studies are further categorized into the areas of segmentation, synthesis, radiomics, and real time MRI. Finally, clinical implications, current challenges, and future directions are discussed.

## 1 INTRODUCTION

Recent innovations in magnetic resonance imaging (MRI) and deep learning are complementary and hold great promise for improving patient outcomes. With the advent of the Magnetic Resonance Imaging Guided Linear Accelerator (MRI-LINAC) and MR-guided radiation therapy (MRgRT), MRI allows for accurate and real-time delineation of tumors and organs at risk (OARs) that may not be visible with traditional CT based plans.[31] Deep learning methods augment the capabilities of MRI by reducing acquisition times, generating electron density information crucial to treatment planning, and increasing spatial resolution, contrast, and image quality. In addition, MRI auto-segmentation and dose calculation methods greatly reduce the required human effort on tedious treatment planning tasks, enabling physicians to further optimize treatment outcomes. Finally, deep learning methods offer a powerful tool in predicting the risk of tumor recurrence and adverse effects. These advancements in MRI and deep learning usher in the era of fully adaptive radiation therapy (ART) and the MRI-only workflow.[176]

Deep learning methods represent a broad class of neural networks which derive abstract context through millions of sequential connections. While applicable to any imaging modality, these algorithms are especially well suited to MRI due to its high information density.[107] Deep learning demonstrates state of the art performance over traditional hand-crafted and machine learning methods but are computationally intensive and require large datasets. For MRI and other imaging tasks, convolutional neural networks (CNNs), built on local context, have traditionally dominated the field. However, advancements in network architecture, availability of more powerful computers, large high-quality datasets, and increased academic interest have led to rapid innovation. Especially exciting are the rapid adaptation of cutting edge recurrent, attention, and self-attention methods which continue to improve upon and even replace CNNs.

Deep learning techniques can be organized according to their applications in MRgRT in the following groups: segmentation, synthesis, radiomics (classification), and real-time/4D MRI. Segmentation methods automatically delineate tumors, organs at risk (OARs), and other structures. However, deep learning approaches face challenges when adapting to small tumors, multiple organs, low contrast, and differing ground truth contour quality and style. These challenges differ greatly depending on the region of the body, so segmentation methods are primarily organized by anatomical region.[134]

Synthesis methods are best understood by their input and output modalities. Going from MRI to CT, synthetic CT (sCT) provides accurate attenuation information not apparent in MRI, augmenting the information of co-registered CT images. In an MRI-only workflow, sCT avoids registration errors and the radiation exposure associated with traditional CT.[68] In addition, synthetic relative proton stopping power (sRPSP) maps can be generated to directly obtain dosimetric information for proton radiation therapy.[239] The dosimetric uncertainty can be further enhanced with deep learning dose calculation methods which greatly reduce inference time and could yield lower dosimetric uncertainties compared to traditional Monte Carlo (MC) methods. Synthetic MRI (sMRI) generated from CT, is appealing by combining the speed and dosimetric information of CT with MRI’s high soft tissue contrast. However, CT’s lower soft tissue contrast makes this application much more challenging, but sMRI has still found success in improving CT-based segmentation accuracy.[49, 122, 132] Alternatively, there are rich intramodal applications by generating one MRI sequence from another. For example, the spatial resolution of clinical MRI can be increased by predicting a higher resolution image[33, 272] and applying contrast can be avoided with synthetic contrast MRI.[103]

Radiomics represents an eclectic body of works but can be divided into studies which classify structures in an MRI image[254] or prognostic models which use MR images to predict treatment outcomes such as tumor recurrence or adverse effects.[119, 273] Deep learning methods in real-time and 4D MRI overcome MRI’s long acquisition time and the low field strengths of the MRI-LINAC by reconstructing images from undersampled k-space[212], synthesizing additional MRI slices[77], and exploiting periodic motion to improve image quality[63].

In this review, we systematically examine studies that apply deep learning to MRgRT, categorizing them based on their application and highlighting interesting or important contributions. We also discuss future trends in deep learning and MRgRT.

## 2 LITERATURE SEARCH

This systematic review surveys literature which implements deep learning methods and MRI for radiation therapy research. “Deep learning” is defined to be any method which includes a neural network directly or indirectly. These include machine learning models and other hybrid architectures which take deep learning derived features as input. Studies including MRI as at least part of the dataset are included. Studies must list their purpose as being for radiation therapy and include patients with tumors. Studies on immunotherapy and chemotherapy without radiation therapy are excluded. Conference abstracts and proceedings are excluded due to an absence of strict peer review.

**Figure 1.**
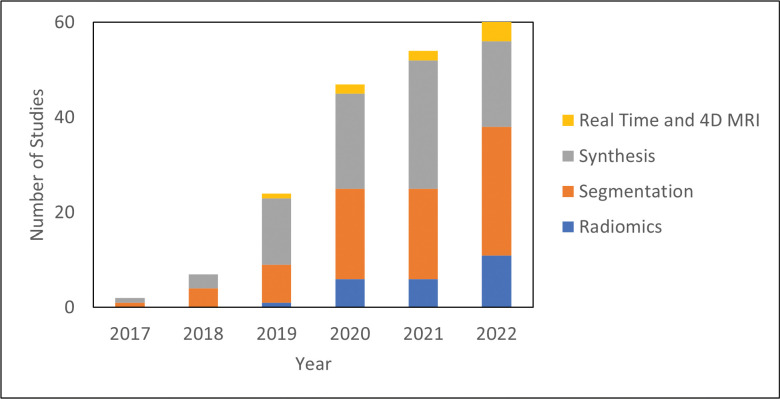
Number of deep learning studies with applications towards MRgRT per year by category.

**Figure 2.**
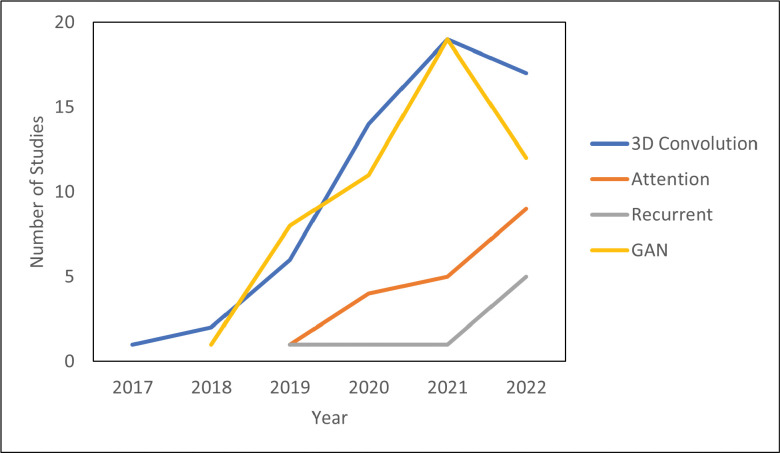
Technical Trends in Deep Learning.

The literature search was performed on Pubmed on December 31, 2022, with the following search criteria in the title or abstract: “deep learning and (MRI or MR) and radiation therapy”. This search yielded 335 results. Of these results, 197 were included based on manual screening using the aforementioned criteria. 78 were classified as segmentation, 81 as synthesis, 24 as radiomics (classification), and 14 as real-time or 4D MRI. There is inevitably some overlap in these categories. In particular, studies which use sMRI for the purposes of segmentation are classified as synthesis and papers which deal with real-time or 4D MRI are placed in Section 6: Real-Time and 4D MRI. Figure 1 shows the papers sorted by category and year. Compared to other review papers, this review paper is more comprehensive in its literature search and is the first specifically on the topic of deep learning in MRgRT. In addition, this work uniquely focuses on the underlying deep learning methods as opposed to their results. Figure 2 shows technical trends in deep learning methods implementing 3D convolution, attention, recurrent, and GAN techniques.

## 3 IMAGE SEGMENTATION

Contouring (segmentation) in MRgRT is the task of delineating targets of interest on MR images which can be broadly divided into distinct categories: contouring of organs at risk and other anatomical structures expected to receive radiation dose and contouring of individual tumors. Contouring is typically performed by dosimetrists, physicists, and physicians. Both tumor and multi-organ segmentation suffer from intra- and inter- observer variability.[58] MRI does not capture the true extent of the tumor volume, as well as poorly defined boundaries and similar structures like calcifications lead to institutional and intra-observer variability. Physician contouring conventions and styles further complicate the segmentation task and lead to inter-observer variability.[8, 10] Multi-organ segmentation is mostly challenged by the large number of axial slices and OARs which make the task tedious and prone to error. Automated solutions to MRI segmentation have been proposed to reduce physician-workload and provide expert-like performance.

Since the application of CNNs to MRI-based segmentation in 2017[155], fully convolutional networks (FCNs) have outperformed competing atlas-based and hand-crafted auto-segmentation methods, often matching the intra-observer variability among physicians[19]. FCNs employ convolutional layers which are trained to detect patterns in either nearby voxels or feature maps output from previous convolutional layers. In contrast with traditional CNNs, FCNs forgo densely connected layers. This design choice enables voxel-wise segmentation, allows for variable sized images, and reduces model complexity and training time. Different types of convolutions include atrous and separable convolutions. Atrous convolutions sample more sparsely to gain a wider field of view and can be mix-and-matched to capture large and small features in the same layer. Separable convolutions divide a 2D convolution into two 1D convolutions to use fewer parameters for similar results. By connecting multiple convolutional layers together with non-linear activation functions, larger and more abstract regions of the input image are analyzed to form the encoder. For pixelwise segmentation, the final feature map is expanded to the original image resolution through a corresponding series of transposed convolutional layers forming the decoder. All FCNs include pooling layers to conserve computational resources whereby the resolution of feature maps is reduced by choosing the largest (max-pooling) or average local pixel.[3]

To evaluate performance, various evaluation metrics are employed with the Dice similarity coefficient (DSC) being the most prevalent. The DSC is defined in equation 1 (Eq 1) as the overlap between the ground truth physician contours and the predicted algorithmic volumes with a value of 0 corresponding to no overlap and 1 corresponding to complete overlap. Mathematically, it is defined as follows where VOLGT is the ground truth volume and VOLPT is the predicted volume[146]:

(1) $$DSC=\frac{2|VOLGT\;\wedge \;VOLPT|}{\left|VOLGT|\;+\;|VOLPT\right|}$$

Additional metrics include the Hausdorff distance[146] which measures the farthest distance between two points of the ground truth and algorithmic volumes, volume difference[15], which is simply the difference in volumes, and the Jaccard Index[52], which is similar to the DSC and measures the overlap between VOLPT and VOLGT relative to their combined volumes. A discussion of these metrics is found in Müller et al.[177] However, performance between datasets must be evaluated with caution due to high inter-observer variation between physicians and dataset quality.

The properties of MRI datasets have driven innovation. Multiple MRI sequences, with and without contrast, are often available. To capture all data, the different sequences are co-registered and input as multiple channels yielding multiple segmentations. These segmentations are combined to produce a final segmentation using an average, weighted average, or more advanced method. To account for MRI’s high through-plane resolution relative to its in-plane resolution, 3D convolutional layers are often utilized to capture features not apparent in 2D convolution. However, 3D convolutions are computationally expensive, so numerous 2.5D architectures have been proposed.[93, 241, 257] In a 2.5D architecture, adjacent MRI slices are input as channels, and 2D convolutions are performed. It is also common to see new papers forgo the 3D convolution to save resources for new computationally intense methods. An unfortunate fact is that high-quality MRI datasets are often small. To remedy this, data augmentation methods such as rotating and flipping the MR images are ubiquitous. In addition, the generation of synthetic images to increase dataset size and generalizability is an exciting field of research.[11] Public datasets and competitions have also helped in this regard. For example, the Brain Tumor Segmentation Challenge (BraTS) dataset[166], updated since 2012, has been a primary contributor to brain segmentation progress, spawning the popular DeepMedic framework[112]. Another approach for small datasets is transfer learning. In transfer learning, a model is trained on a large dataset, and then retrained on a smaller dataset with the idea that many of the previously found features are transferable.[275]

Advances from the field of natural language processing (NLP) have had a tremendous impact on segmentation tasks. Recurrent neural networks (RNNs) are defined by the output of their node being connected to the input of their node. To avoid an infinite loop, the output is only allowed to connect to its input a set number of times. This property allows for increased context and the ability to handle sequential data which is especially important in language translation. Applied to CNNs, each recurrent convolutional layer (convolution + activation function) is preformed multiple times which creates a wider field of view and more context with each subsequent convolution. However, recurrent layers can suffer from a vanishing gradient problem. Long short-term memory blocks (LSTM) solve this by adding a “forget” gate which forgets irrelevant information. In addition, LSTMs are more capable of making long range connections. Similar to the LSTM gate, the gated recurrent unit (GRU) has an update and reset gate which decide which information to pass on and which to forget. Both LSTM and GRU also have bidirectional versions which pass information forward and backwards.[36, 154] Relative performance between the LSTM and GRU gates are situational with the GRU gate being less computationally expensive.[252]

A major issue faced in MRI-segmentation can be characterized as “the small tumor problem”. Small structures like tumors or brachytherapy fiducial markers represent a small fraction of the total MRI volume, where CNNs can struggle to find them or be confused by noise. Further exacerbating the problem is that applying a deep CNN to whole MR images consumes extensive computational resources, so the MRI must be downsampled. In this case, the downsamplying is very likely to cause small tumors to be missed entirely. One of the simplest ways to improve performance is to alter the loss function. Standard loss functions are cross-entropy and dice loss which seek to maximize voxel wise classification accuracy and overlap between the predicted and ground truth contours, respectively. These can be modified to achieve higher sensitivity to small structures at the expense of accuracy. Focal loss is the cross-entropy loss modified for increased sensitivity[149] and Tversky loss does the same for the dice loss.[202] In addition, borders of the contours are the most important part of the segmentation, so boundary loss functions seek to improve model performance by placing increased emphasis on regions near the contour edge.[85, 224] Another approach to solve the problem, albeit at the expense of long-range context, is with two stage networks. In the first stage, regions of interest (ROIs) are identified, and target structures are then contoured in the ROIs in the second stage. Notable efforts include Mask R-CNN[83] and Retina U-Net[97] which implement convolution-based ROI sub-networks with advanced correction algorithms. Seqseg instead replaces the correction algorithms with a reinforcement learning based model.[224] An agent is guided by a reward function to iteratively improve the conformity of the bounding box. Seqseg reported comparable performance with higher bounding box recall and intersection over union (IoU) compared to Mask R-CNN.

Related developments from NLP are the concepts of attention and the transformer. In terms of MRI, attention is the idea that certain regions of the MRI volume are more important to the segmentation task and should have more resources allocated to them. ROI schemes can then be defined as a form of hard attention by only considering the region around a tumor. A version of soft attention would weight the region around the tumor heavily and process the information in high resolution but also give a smaller weighting to nearby organs and process it in lower resolution.[214] In practice, attention modules include a fully connected feedforward neural network to generate weights between a feature map of the encoder and a shallower feature map in the decoder. These weights are improved upon through backpropagation of the entire network to give higher representational power to contextually significant areas of the image. This fully connected network can also be replaced with other models such as the RNN, GRU, or LSTM.[269] If the same feature map is compared with itself, this is called self-attention and is the basis for the transformer architecture.[233] The transformer can be thought of as a global generalization of the convolution and can even replace convolutional layers. The advantages of the transformer are explicit long-range context and the transformer’s multi-head attention block allows for attention to be focused on different structures in parallel. However, transformers require more data to train and can be very computationally expensive. Such computational complexity can be remedied by including convolutional layers in hybrid CNN-transformer architectures[137], by making long range connections between voxels sparse,[30] or by implementing more efficient self-attention models like FlashAttention.[42]

From the field of neuroscience, deep spiking neural networks (DSNNs) attempt to more closely model biological neurons by connecting neurons with asynchronous time dependent spikes instead of the continuous connections between neurons of traditional neural networks. Potential advantages include lower power use, real-time unsupervised learning, and new learning methods. However, these advantages are only fully realized with special neuromorphic hardware, are difficult to train, and currently lag conventional approaches. For these reasons, they are currently only represented by one paper in this review.[1]

Many new models for MRI segmentation have been created by modifying U-Net. U-Net derives its name from its shape which features convolutional layers in the encoder and transposed convolutional layers in the decoder. Its main innovation, however, is its long-range skip connections between the encoder and decoder. Dense U-Net densely connects convolutional layers in blocks[18], ResU-Net includes residual connections[45], Retina U-Net is a two-stage network, RU-Net includes recurrent connections, R2U-Net adds residual recurrent connections[2]. Attention modules have also been added at the skip connections.[185, 262] The aforementioned networks were all designed with 2D convolutions but can be modified to include 3D convolutions. Both V-Net[170] and nnUNet[94] were designed with 3D convolutional layers with nnUNet additionally automating preprocessing and learning parameter optimization. Pix2pix uses U-Net as the generator with a convolutional discriminator (PatchGAN)[95]. Other state-of-the-art architectures include Mask R-CNN, DeepMedic, and DeepLabV3+.[25] Mask RCNN is a two-stage network with a ResNet backbone. Mask Scoring RCNN (MS-RCNN) improves upon Mask R-CNN by adding a module which penalizes ROIs with high classification accuracy but low segmentation performance[91]. DeepMedic, designed for brain tumor segmentation, is an encoder-only CNN which inputs a ROI and features two independent row-resolution and normal resolution channels. These channels are joined in a fully connected convolutional layer to predict the final segmentation. The convolutions in the encoder-only style reduce the final segmentation map dimensions compared to the original ROI (25×25×25 vs 9×9×9 voxels). DeepLabV3+ leverages residual connections and multiple separable atrous convolutions. Xception improves upon the separable convolution by reversing the order of the convolutions and including ReLU blocks after each operation for non-linearity.[32]

### 3.1 Brain

Largely unaffected by patient motion and comprised of detailed soft tissue structures, the brain is an ideal site to benchmark segmentation performance for MRI and represents the dominant category in MRI segmentation research. Unique to brain MRI preprocessing is skull stripping, where the skull and other non-brain tissue are removed from the image. This can significantly improve results, especially for networks with limited training data.[111] Shown in table 1, the majority of the studies focus on segmenting different brain tumors such as glioma, Glioblastoma Multiforme (GBM), and metastases. A small minority of studies focuses on OARs like the hippocampus. Advancements in brain segmentation have come, in large part, from the yearly Multimodal Brain Tumor Image Segmentation Benchmark (BraTS) challenge, which includes high quality T1-weighted (T1W), T2-weighted (T2W), T1-contrast (T1C), and T2 -Fluid-Attenuated Inversion Recovery (FLAIR) sequences with the purpose of segmenting the whole tumor (WT), tumor core (TC), and enhancing tumor (ET) volumes. The WT is defined as the entire spread of the tumor visible on MRI; The ET is the inner core which shows significant contrast compared to healthy brain tissue, and the TC is the entire core including low contrast tissue. The most popular architectures are DeepMedic, created for the BraTS challenge, and U-Net.

Notable efforts in the BraTS challenge include Momin et al achieving an exceptional WT dice score of .97 ±.03 with a Retina U-Net based model and mutual enhancement strategy. In their model, Retina U-Net finds a ROI and segments the tumor. This feature map is fed into the classification localization map (CLM module) which further classifies the tumor into subregions. The CLM shares the encoding path with a segmentation module, so classification and segmentation share information and are improved iteratively.[173] Huang et al focuses on correctly segmenting small tumors. Based on DeepMedic, the method incorporates a prior scan and custom loss function, the volume-level sensitivity–specificity (VSS), which rates and significantly improves the metastasis sensitivity and specificity to segment small brain metastases.[89] Another paper improves small tumor detection by 2.5 times compared to the standard dice loss by assigning a higher weight to small tumors.[24] Lee et al takes the novel approach of using standard dice loss for the first 40 epochs and changes to Tversky loss for the final 20 epochs to specify sensitivity and specificity. [130] Both Tian et al[228] and Ghaffari et al[72] utilize transfer learning datasets to cope with limited data. Pan et al includes a two-stage U-Net model with residual and attention blocks. [193] Ahmadi et al achieves competitive results in the BraTS challenge with a DSNN. [1]

### 3.2 Head and Neck

The head and neck (HN) region contains many small structures, making high-resolution and high-contrast imaging of great importance. MRI is especially preferred over CT imaging for patients with amalgam dental fillings due to the metallic content that can cause intense streaking artifacts on CT.[46] In addition, MRI is the standard of care for nasopharyngeal carcinoma (NPC), leading to significant research attention on auto-segmentation algorithms for HN MR images. Other research efforts include segmentation of oropharyngeal cancer, glands, and lymph nodes in the American Association of Physicists in Medicine (AAPM)’s RT-MAC challenge[20], as well as multi-organ segmentation.

Notable efforts include the two-stage multi-channel Seqseg architecture for NPC segmentation.[147] Seqseg uses reinforcement learning to refine the position of the bounding box, implements residual blocks, recurrent channel and region-wise attention, and a custom loss function that emphasizes segmentation of the edges of the tumor. Outierial et al[200] improves the dice score by 0.10 with a two-stage approach compared to single-state 3D U-Net for oropharyngeal cancer segmentation. For multiparametric MRI (mp-MRI), Deng et al[43] concludes that the union output from T1W and T2W sequences has similar performance to T1C MRI, suggesting that contrast may not be necessary for NPC segmentation. Similarly, Wahid et al[234] found that T1W and T2W sequences significantly improve performance, but dynamic contrast enhanced MRI (DCE) and diffusion weighted imaging (DWI) have little effect. Interesting approaches to gland and lymph node segmentation came out of the AAPM’s RT-MAC challenge, with Kawahara et al’s[115] 2.5D GAN and Korte et al[127] employing a 2-stage architecture. The first stage segments the OARs in low resolution to create a bounding box, followed by U-Net segmenting the ROI in high resolution. Jiang et al segments the parotid glands using T2W MRI and unpaired CT images with ground truth contours. First, sMRI is generated from the CT volumes using a GAN. In the second step, U-Net generates probabilistic segmentation maps for both the sMRI and MRI based on the CT ground truth contours. These maps, along with sMRI and MRI data, are then input into the organ attention discriminator, which is designed to learn finer details during training, ultimately producing the final segmentations.[104]

### 3.3 Abdomen, Heart, And Lung

In contrast to the brain, the abdomen is susceptible to respiratory and digestive motion of the patient often leading to poorly defined boundaries. While motion management techniques like patient breath-hold and not eating or drinking before treatment can mitigate these effects, the long acquisition time of MRI will inevitably lead to errors. Often physicians must rely on anatomical knowledge to deduce the boundaries of OARs. This makes segmentation challenging for CNN-based architectures which build from local context. In addition, registration errors make including multiple sequences impractical. OARs segmented in the abdomen include the liver, kidneys, stomach, bowel, and duodenum.

The liver and kidneys are not associated with digestion and are relatively stable while the stomach, bowel, and duodenum are considered unstable. The duodenum is the most difficult for segmentation algorithms due to its small size, low contrast, and variability in shape. In addition, radiation induced duodenal toxicity is often dose-limiting in dose escalation studies making accurate segmentation of high importance.[120] Similar problems occur in the heart and lung because of their periodic motion with the lung being particularly challenging since it is filled with low-signal air. However, MR segmentation of cardiac subregions have shown growing interest as these are not visible on CT and have different tolerances to radiation.[219]

The results are summarized in Table 3. Due to the large number of organs segmented in several of these studies, only the stomach and duodenum dice scores are reported to establish how the algorithms handle unstable organs. Zhang et al[161] generates a composite image from the current slice, prior slice, and contour map to pre-dict the current segmentation with U-Net. Luximon et al[161] takes a similar approach by having a phy-sician contour every 8th slice. These contours are then linearly interpolated and improved upon with a 2D Dense U-Net. The remaining studies do not require previous information and struggle to segment the duodenum. Ding et al[47] improves upon a physician-defined acceptable contour rate by up to 39% with an active contour model. A 3D Dense U-Net with sequential refinement networks is included in Fu et al[66]. Morris et al segments heart substructures with a 2 channel 3D U-Net.[175] Wang et al segments lung tumors with high accuracy relying on segmentation maps from previous weeks with the aim of adaptive radiation therapy (ART).[237] An addition study by the same group feeds the features from the CNN into a GRU based RNN to predict tumor position over the next 3 weeks. Attention is included to weigh the importance of the prior weeks’ segmentation maps.[236]

### 3.4 Pelvis

The anatomy of the pelvis allows both external beam radiation therapy (EBRT) and brachytherapy approaches for radiation therapy. Therefore, MRI segmentation studies have proposed methods to contour fiducial markers and catheters for cervical and prostate therapy, as well as tumors and OARs. However, a current challenge is that fiducials and catheters are designed for CT and are not optimal for MRI segmentation. For example, in prostate EBRT, gold fiducial markers localize the prostate with high contrast and correct for motion. However, metal does not emit a strong signal on MRI, so fiducials on MRI are characterized by an absence of signal, which can be confused with calcifications. Despite this, MRI is enabling treatments with higher tumor conformality. For instance, the gross tumor volume (GTV) of prostate cancer is not well delineated on CT but is often visible on MRI. In addition, the prostate apex is significantly clearer on MRI.[235] MRI-based focal boost radiation therapy, in addition to a single dose level to the whole prostate, escalates additional dose to the GTV to reduce tumor recurrence.[121]

**Figure 3.**
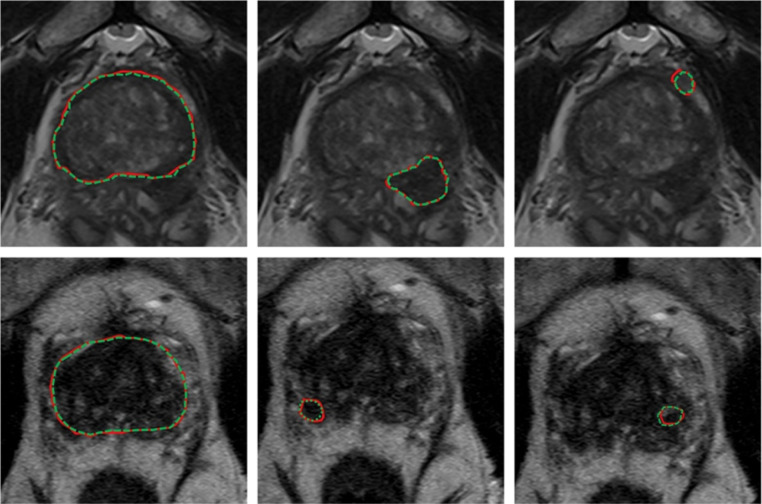
Expert (red) versus proposed auto-segmented (green dashed) prostate and DIL contours on axial MRI. From left to right: prostate manual and auto-segmented contours overlaid on MRI, and two DIL manual and auto-segmented contours overlaid on MRI. The upper and lower rows are representative of two patients. Reprinted by permission from John Wiley and Sons: Medical Physics, MRI-based prostate and dominant lesion segmentation using cascaded scoring convolutional neural network by Eidex et al[52] © 2022.

Table 4 shows relevant auto-segmentation techniques applied to the pelvic region. Shaaer et al[208] segments catheters with a T1W and T2W MRI-based U-Net model and takes advantage of catheter continuity to refine the contours in post processing. Zabihollahy et al[260] creates an uncertainty map of cervical tumors by retraining the U-Net model with a randomly set dropout layer. This technique is called Monte Carlo Dropout (MCDO). Cao et al[19] takes pre-implant MRI and post-implant CT as input channels to their network. After preforming intra-observer variability analysis, they achieve performance more similar to a specialist radiation oncologist for cervical tumors in brachytherapy than a non-specialist. Eidex et al[52] segments dominant intraprostatic lesions (DILs) and the prostate for focal boost radiation therapy with a Mask R-CNN based architecture. Sensitivity is found to be an important factor in evaluating model performance because weak models can appear strong by missing difficult lesions entirely. Figure 3 shows an example of automatic contours of the prostate and DIL on T2w MRI which would not be visible on CT. STRAINet[180] realizes exceptional performance by utilizing a GAN with stochastic residual and atrous convolutions. In contrast with standard residual connections, each element of the input feature map which does not undergo convolution has a 1% chance of being set to zero. Singhrao et al[215] implements a pix2pix architecture for fiducial detection achieving 96% detection with the misses caused by calcifications.

## 4 IMAGE SYNTHESIS

Synthesis is an exciting field of research, defined as translating one imaging modality into another. Benefits of synthesis include avoiding potential artifacts, reducing patient cost and discomfort, and avoiding radiation exposure.[243] In addition, utilizing multiple modalities introduces registration errors which can be avoided with synthetic images. Current methods in MRgRT include synthesis of sCT from MRI, sMRI from CT, and relative proton stopping power images from MRI. Other areas of synthesis research include creating higher resolution MRI (super-resolution) and predicting organ displacement based on periodic motion in 4D MRI. Segmentation can also be thought of as a special case of synthesis because the input MRI is translated into voxel-wise masks which assume discrete values according to their class. The distinction between synthesis and segmentation is particularly muddied when the segmentation ground truth is from a different imaging modality.[207]

Synthesis architectures are fundamentally interchangeable with segmentation architectures but have diverged in practice. For example, U-Net, described in detail in Section 3, is the predominant backbone in both areas. However, synthesis models require that the entire image be translated, so that they do not include two-stage architectures and are dominated by generational adversarial network (GAN) - based architectures. The GAN is comprised of a CNN or self-attention-based generator which generates synthetic images. The generator competes with a discriminator which attempts to correctly classify synthetic and real images. As the GAN trains, a loss function is applied to the discriminator when it mislabels the image, whereas a loss function is applied to the generator when the discriminator is correct. The model is ideally considered trained once the discriminator can no longer correctly identify the synthetic images. Conditional GANs (cGANs) expand on the standard GAN by also inputting a vector with random values or additional information into both the generator and discriminator.[171] In the case of MRI, the values of the vector can correspond to the MRI sequence type and clinical data to account for differences in patient population and setup. The CycleGAN adds an additional discriminator and generator loop.[274] For example, an MRI would be translated into a sCT. The sCT would then be translated into a sMRI. Since the input is ultimately tested against itself, this allows for training with unpaired data. The need for co-registration is eliminated but requires significantly more data to achieve comparable results with paired training.

Despite their success, GANs can be unstable during training and struggle in difficult synthesis problems. One way to improve its performance is with the Wasserstein GAN (WGAN)[6]. Instead of the discriminator classifying the images as real or fake, the WGAN measures the probability distributions of the real and fake images and finds the distance between them in the form of the Wasserstein distance. The discriminator attempts to maximize this distance while the generator attempts to minimize it. The WGAN approach often improves stability and performance. Although not limited to WGANs, spectral normalization is often included which constricts the training weights of the discriminator such that the gradient cannot explode. Another approach, claiming better performance than the WGAN, is the relativistic GAN (RGAN)[108]. The RGAN claims that the generator should, in addition to increasing the probability that synthetic images appear realistic, increase the probability that real images appear fake to the discriminator. Without this condition, the discriminator will conclude that every image it comes across is real in the late stages of training with a well-trained generator. This goes against the priori knowledge that half of the images are fake. A standard GAN can be converted to a RGAN by modifying its loss function.

### 4.1 MRI-Based Synthetic CT

MRI-based sCT is the most extensively researched and influential application of synthesis models in radiation therapy. While MR images provide excellent soft tissue contrast, they do not contain the necessary attenuation information for dose calculation that is embedded in CT images. Owing to this limitation, CT has traditionally been the workhorse for treatment planning while MRI has been relegated to diagnostic applications. However, CT suffers from lower soft tissue contrast and imparts a non-negligible radiation dose, especially for patients receiving standard fractionated image guided radiation therapy (IGRT). In addition, metallic materials found in dental work and implants can lead to severe artifacts in CT, reducing the quality of the treatment plan. By augmenting CT with sCT, these problems can be avoided. Furthermore, according to the “As Low As Reasonably Achievable” (ALARA) principle, the replacement of CT with sCT for an MRI only workflow could be justified with its high accuracy, especially in radiosensitive populations like pediatric patients.[184, 242]

Calculation of dose distribution using MRI-based sCT can be enhanced by replacing traditional Monte Carlo simulation (MC) techniques with deep learning. MC accurately predicts the dose distribution based on physical principles, including the electron return effect (ERE), which adds additional dose to boundaries with different proton densities in the presence of a magnetic field. However, the technique can be extremely slow, as it relies on randomly generating paths of tens of thousands of particles. The higher number of particles reduces dosimetric uncertainty. This problem is particularly noticeable in proton therapy, where MC or pencil beam algorithm (PBA) calculations can take several minutes on a CPU, and it can take hours to optimize a single treatment plan.[192] As a result, compromises must be made in clinical practice between dosimetric uncertainty, MC run time, and treatment plan optimization. Deep learning methods show exceptional potential to improve upon MC dose calculation models. Once trained, deep learning algorithms take only a few seconds to synthesize a dose distribution. In addition, they can be trained on extremely high accuracy MC generated dose distributions that would be impractical in everyday clinical practice.

The primary challenge to sCT methods is the accurate reconstruction of bone and air, due to their low proton density and weak signal. This can make it difficult for sCT to distinguish between the two, leading to large errors. In addition, further complicating the issue is that bone makes up a small fraction of the patient volume in radiation therapy tasks or applications which is similar to the “small tumor problem” seen in segmentation. Other issues that can arise include small training sets, misalignment between CT and MRI, and causes of high imaging variability such as intestinal gas.

To evaluate sCT performance, various metrics are used to compare voxel values between the ground truth CT and sCT. The most common metric is the mean absolute error (MAE)[140, 276] which is reported in tables 5 and 6 if available. The MAE is defined below in Eq 2, where xi and yi are the corresponding voxel values of the CT and sCT, respectively, and n is the number of voxels.


(2) $$MAE=\Sigma_{i=1}^{n}\left|y_{i}-x_{i}\right|/n$$

The MAE is typically reported in Hounsfield units (HU) but can also be dimensionless if reported with normalized units. Other common metrics in literature are the mean error[129], which forgoes the absolute value in MAE, the mean squared error (MSE)[258], which substitutes absolute value for the square, and the Structural Similarity Index (SSIM), which varies from −1 to 1 where −1 represents extremely dissimilar images and 1 reperesents identical images.[204] A full discussion of these metrics can be found in Necasova et al.[179] Since sCT is primarily intended for treatment planning, dosimetric quantities which measure the deviation between CT- and sCT-derived plans are often reported. One of the most common metrics is gamma analysis. Repurposed as a metric to compare treatment plan dose to actual dose on LINACs, gamma analysis looks at each point on the dose distribution and evaluates if the acceptance criteria are met. The American Association of Physicists in Medicine (AAPM) Task Group 119 recommends a low dose threshold of 10%, meaning that points which receive less than 10% of the maximum dose are excluded from the calculation. Other metrics include the mean dose difference and the minimum dose delivered to 95% of the clinical treatment volume (D95) difference.

**Figure 4.**
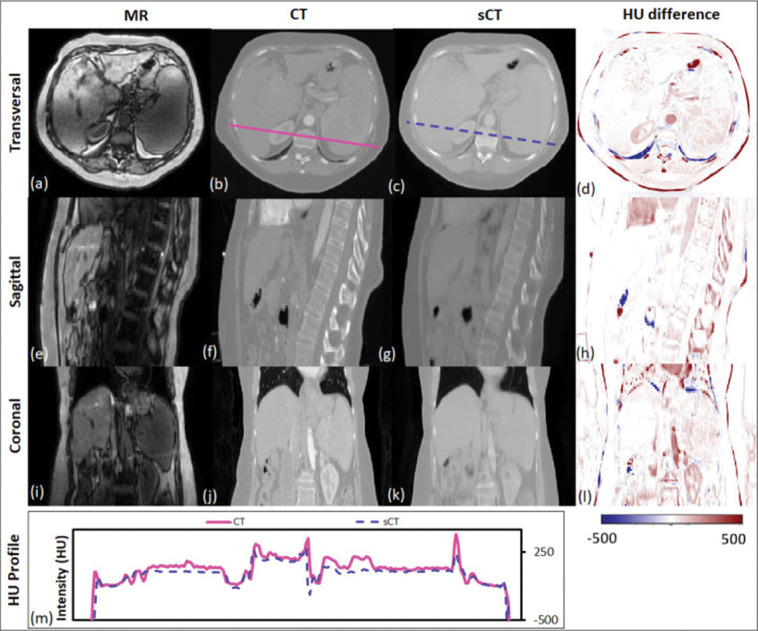
Traverse, sagittal, and coronal images of a representative patient. MRI, CT and sCT images and the HU difference map between CT and sCT are presented. The CT (solid line) and sCT (dashed line) voxel-based HU profiles of the traverse images are compared in the lowermost panel. Reprinted by permission from British Journal of Radiology, MRI-based treatment planning for liver stereotactic body radiotherapy: validation of a deep learning-based synthetic CT generation method by Liu et al.[156]© 2019.

Sampling notable MRI-based sCT works for photon radiation therapy, several take advantage of cGANs to include additional information. Liu et al improves upon the CycleGAN by including a dense block, which captures structural and textural information and better handles local mismatc¬hes between MRI and ground truth CT images. In addition, a compound loss function with adversarial and distance losses improves boundary sharpness. An example patient is shown in Figure 4.[156] Hsu et al proposes a 2.5D method by training a pix2pix-based architecture with axial, sagittal, and coronal MRI slices.[86] A conditional CycleGAN in Boni et al passes in MR manufacturer information and achieves good results despite using unpaired data and different centers for their training and test sets.[17] Many studies also experiment with multiple sequences. Massa et al trains a U-Net with Inception-V3 blocks on 1.5T T1W, T2W, T1C, and FLAIR sequences separately and finds no statistical difference.[165] However, Koike et al uses multiple MR sequences for sCT generation employing a cGAN to provide better image quality and dose distribution results compared with those from only a single T1W sequence.[126] Dinkla et al finds that sCT removes dental artifacts.[48] Farjam et al implements a custom loss function, which reweights contributions from bone.[56] Wang et al uses a 3D pix2pix architecture, averaging results from 4×4×4, 6×6×6, and 8×8×8 filters to improve their results.[240] Instead of using a GAN, Li et al simulates one with a fixed discriminator by pretraining a VGG16 loss network to discriminate between sCT and CT[142]. Reaungamornrat et al decomposes features into modality specific and modality invariant spaces between high- and low-resolution Dixon MRI with the Huber distance.[197] In addition, separable convolutions are used to reduce parameters, and a relativistic loss function is applied to improve training stability. Finally, Zhao et al represents the first MRI-based sCT paper to implement a hybrid transformer-CNN architecture outperforming other state-of-the-art methods. Their method implements a conditional GAN. The generator consists of CNN blocks in the shallow layers to capture local context and save computational resources, while transformers are used in deeper layers to provide better global context.[267]

Generating sCTs from MRI for the purposes of proton therapy is not fundamentally different from the process for photon therapy. However, proton therapy takes advantage of the Bragg peak, which concentrates the radiation in a small region to spare healthy tissue. While this is beneficial, this puts a tighter constraint on sCT errors. Another difference is that sCT images must first be converted to relative proton stopping power maps before they can be used in treatment planning. Therefore, directly generating synthetic proton relative stopping power (sRPSP) maps instead of sCT would be ideal. Boron therapy is a form of targeted radiation therapy in which boronated compounds are delivered to the site of the tumor and irradiated with neutrons. The boron undergoes a fission reaction, releasing alpha particles that kill the tumor cells. However, the targeting mechanism typically relies on targeting cancer cells’ high metabolic rate. Epidermal tissue that also has a high metabolic rate uptakes boron, making skin dose an important concern in boron therapy. Therefore, methods for generating sCT images for boron therapy should emphasize accurate reconstruction around the skin. Shown in Table 6, many methods show high dosimetric accuracy for proton therapy. Liu et al develops a conditional cycleGAN to synthesize both high and lower energy CT. Multiple loss functions are also used to accurately classify and recreate the sCT.[152] Wang et al creates the first synthetic relative proton stopping power maps from MRI with a cycleGAN and loss function to take advantage of paired data. Their method achieves an excellent MAE of 42 ± 13 HU, but struggles with dosimetric accuracy.[239] Maspero et al achieves a 2%/2mm gamma pass rate above 99% for proton therapy by averaging predictions from three separate GANs trained on axial, sagittal, and coronal views, respectively.[164] Replacing traditional MC dose calculation methods, Tsekas et al generates VMAT (volumetric modulated arc therapy) dose distributions in static positions with sCT. Additionally, parameters include a mask of the tissue exposed to the beam, the distance from LINAC source, the distance from central beam, and the radiological depth.[232] These parameters are input into a 3D U-Net, significantly increasing processing speed. Finally, SARU, a self-attention Res-UNet, lowers skin dose for boron therapy, achieving better results than the pix2pix method.[268]

### 4.2 CT and CBCT-Based Synthetic MRI

Generating sMRI from CT leverages MRI’s high soft tissue contrast for improved segmentation accuracy and pathology detection for CT-only treatment planning. In addition, the ground truth X-ray attenuation information is maintained compared to an MRI-only workflow. Cone beam CT (CBCT) is primarily used for patient positioning before each fraction of radiation therapy. Kilovoltage (kV) and megavoltage (MV) energies are standard in CBCT with kV images providing superior contrast and MV images providing superior tissue penetration. However, noise and artifacts can often reduce CBCT image quality.[217] Generating CBCT-based sMRI can yield higher image quality and soft-tissue contrast while also retaining CBCT’s fast acquisition speed. CT and CBCTs’ rapid acquisition time can make it preferable over MRI for patients with claustrophobia during the MR simulation or for pediatric patients who would require additional sedation. In addition, MRI is not suitable for patients with metal implants such as pacemakers. However, sMRI is significantly more challenging to generate compared to sCT. This is primarily due to the recovery of soft tissue structures visible only in MRI. For this reason, sMRI is often used to improve segmentation results in CT and CBCT. However, some studies report direct use of sMRI for segmentation. Since MRI intensity is only relative and not in definitive units like CT, MAE is much less meaningful than other metrics. Therefore, peak signal to noise ratio (PSNR) is preferentially reported.[22]

**Figure 5.**
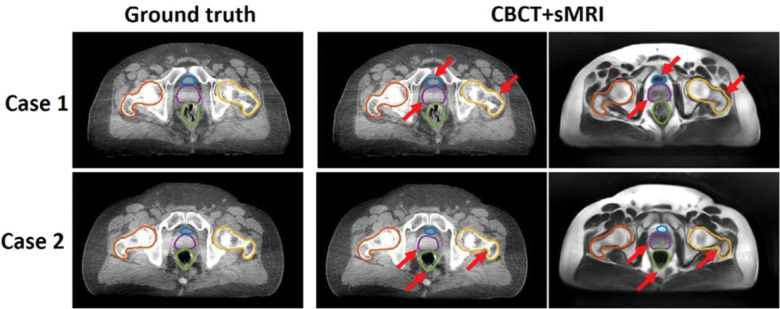
Contours of segmented pelvic organs for two representative patients. Ground truth contours are overlaid onto CBCT. The predicted contours of the proposed method are overlaid on CBCT and sMRI. Red arrows highlight regions in which CBCT and sMRI provide complementary information for bony structure and soft tissue segmentation. Reprinted by permission from John Wiley and Sons: Medical Physics, Pelvic multi-organ segmentation on cone-beam CT for prostate adaptive radiotherapy by Fu et al.[67] © 2020.

For CT-based sMRI, Dae et al implements a cycleGAN for sMRI synthesis with dense blocks in the generator. The sMRIs are input into MS-RCNN improving segmentation performance.[39] Kalantar et al compares U-Net, U-Net++, and cycleGAN, concluding that cycleGAN preforms the best.[110] Lei et al incorporates dual pyramid networks to extract features from both sMRI and CT and includes attention to achieve exceptional results.[133] BPGAN synthesizes both sMRI and sCT bidirectionally with a cycleGAN. Pathological prior information, an edge retention loss, and spectral normalization improve accuracy and training stability.[249] Both CBCT-based sMRI studies, from Emory’s Deep Biomedical Imaging Lab, significantly improve CBCT segmentation results. In their first paper, Lei et al generates sMRI with a CycleGAN, then inputs this into an attention U-Net.[132] Fu et al makes additional improvements by generating the segmentations with inputs from both CBCT and sMRI and also including additional pelvic structures. Example contours overlaid onto CBCT and sMRI are shown in Figure 5.[67]

### 4.3 Intramodal MRI Synthesis and Super Resolution

It can be beneficial to synthesize MRI sequences from other MRI sequences. Intra-modal applications include generating synthetic contrast MRI to prevent the need for injected contrast, super-resolution MRI to improve image quality and reduce acquisition time, and synthetic 7T MRI due to its lack of widespread availability and improve spatial resolution and contrast.[210] To reduce complexity and cost, a potential approach to radiation therapy is to rotate the patient instead of using a gantry. However, the patient’s organs deform under gravity, requiring multiple MRIs at different angles for MRgRT. MR images of patients rotated at different angles can better enable gantry free radiation therapy. In this section, synthesis studies which synthesize other MRI sequences are discussed.

Preetha et al synthesizes T1C images with a multi-channel T1W, T2W, and FLAIR MRI sequences using the pix2pix architecture.[103] A cycleGAN with a ResUNet generator is trained to generate lateral and supine MR images for gantry-free radiation therapy.[27] ResUNet is also implemented to generate ADC uncertainty maps from ADC maps for prostate cancer and mesothelioma.[278] Studies designed explicitly for super-resolution include Chun et al and Zhao et al. In the former study, a U-Net based denoising autoencoder is trained to remove noise from clinical MRI. Since there is a limited number of paired low-resolution and high-resolution MR images, a CNN is trained to downsample high resolution data from this dataset. Finally, a GAN utilizing both residual and skip connections synthesizes the high resolution MRI with high accuracy.[33] The same architecture is employed in Kim et al[123] for real-time 3D MRI to increase spatial resolution. In addition, dynamic keyhole imaging is formulated to reduce acquisition time by only sampling central k-space data associated with contrast. The peripheral k-space data associated with edges is added from previously generated super-resolution images in the same position.[123] Zhao et al makes use of super-resolution for brain tumor segmentation, increasing the dice score from 0.724 to 0.786 with 4x super resolution images generated from a GAN architecture. The generator has low- and high-resolution paths and dense blocks.[272] Often in clinical practice, the through place resolution is increased to reduce the MRI scan time. Xie et al achieves near perfect accuracy in recovering 1 mm from 3 mm through plane resolution by training parallel CycleGANs which predict the higher resolution coronal and sagittal slices, respectively. These predictions are then fused to create the final 3D prediction.[248] No studies have published yet to synthesize 7T MRI for radiation therapy.

## 5 RADIOMICS (CLASSIFICATION)

Unlike synthesis which maps one imaging modality to another, radiomics extracts imaging data to classify structures or to predict a value. Deep learning applications to MRI-based radiomics often achieve state-of-the-art performance over hand-crafted methods in detection and treatment outcome prediction tasks. Traditional radiomics algorithms apply various hand-crafted matrices based on shape, intensity, texture, and imaging filters to generate features. The majority of these features have no predictive power, and would confuse the model if all were directly implemented. Therefore, an important step is feature reduction which screens out features without statistical significance. Typically, this is done with a regression such as analysis of variance (ANOVA), Least Absolute Shrinkage and Selection Operator (LASSO), or ridge regression. Alternatively, a CNN or other neural network can learn significant features. The advantage of the deep learning approach is that the network can learn any relevant features including handcrafted ones. However, this assumes a large enough dataset which can be problematic for small medical datasets. Hand-crafted features have no such constraint and are easily interpretable. It is often the case that a hybrid approach including both hand-crafted and deep learning features yields the highest performance. Biometric data like tumor grade, patient age, and biomarkers can also be included as features. Once the significant features are found, supervised machine learning algorithms like support vector machines, artificial neural networks, and random forests are employed to make a prediction from these features. Recently, CNNs like Xception and InceptionResNet[221], recurrent neural networks with GRU and LSTM blocks, and transformers have also found favor in this task, as introduced in Section 3. Radiomics can also be done purely with deep learning as it is done with segmentation and synthesis. In this section, we divide the studies into those detecting or classifying objects in the image and studies predicting a value such as the likelihood of distant metastases, treatment response, and adverse effects. While detection is traditionally under the purview of segmentation, the architectures of detection methods and the classification task are in common with other radiomics methods, and so are discussed here.

While radiomics algorithms can excel on local datasets, the main concern for MRI applications is the generalizability of the methods. Variability in MR imaging characteristics such as field strength, scanner manufacturer, pulse sequence, ROI or contour quality, and the feature extraction method can result in different features being significant. This variability can largely be mitigated by normalizing the data to a reference MRI and including data from multiple sources.[35]

Classification accuracy is an appealing evaluation metric due to its simplicity, but accuracy can be misleading with unbalanced data. For example, if 90% of tumors in the dataset are malignant, a model can achieve 90% accuracy by labeling every tumor as malignant. Precision[14], the ratio of true positives to all examples labeled as positive by the classifier, and recall[119], the ratio of true positives to all actual positives, will also both differ if given imbalanced data. The F1 score[80] is defined in Eq 3, ranging from 0 to 1 and combining precision and recall to provide a single metric. A high F1 value indicates both high precision and recall and is resilient towards unbalanced data.


(3) $$F1=\frac{2(Recall\;*\;Precision)}{Recall\;+\;Precision}$$

The most common evaluation metric resistant to unbalanced data is the area under the curve (AUC) of a receiver operating characteristics (ROC) curve[22, 99, 182]. In a ROC curve, the x-axis represents the false positive (FP) rate while the y-axis relates the true positive (TP) rate. In addition, the ROC curve can be viewed as a visual representation to help find the best trade-off between sensitivity and specificity for the clinical application by comparing one minus the specificity versus the sensitivity of the model. The AUC value provides a measurement for the overall performance of the model with a value of 0.5 representing random chance and a value of 1 being perfect classification. If the AUC value is below 0.5, the classifier would simply need to invert its predictions to achieve higher accuracy. It is important to note that all these metrics are for binary classification but are commonly used in multi-class classification by comparing a particular class with an amalgamation of every other category. Finally, the concordance index (C-index) measures how well a classifier predicts a sequence of events and is most appropriate for prognostic models which predict the timing of adverse effects, tumor recurrence, or patient survival times. The C-index ranges from 0 to 1 with a value of 1 being perfect prediction.[82, 160] A full discussion of evaluation metrics for classification tasks is found in Hossin and Suliaman.[84]

### 5.1 Cancer Detection and Staging

Effectively detecting and classifying tumors is vital for treatment planning. Deep learning detection methods supersede segmentation algorithms when the tumors are difficult to accurately segment or cannot easily be distinguished from other structures. In addition, detection models can further improve segmentation results by eliminating false positives. When applied to MRI, detection studies also have the potential to differentiate between cancer types and tumor stage to potentially avoid unnecessary invasive procedures like biopsy.

The majority of works in detection are for brain lesion classification. Chakrabarty et al attains exceptional results in differentiating between common types of brain tumors with a 3D CNN and outperforms traditional hand-crafted methods.[22] Radiation induced cerebral microbleeds appear as small dark spots in 7T time of flight magnetic resonance angiography (TOF MRA) and can be difficult to distinguish from look-a-like structures. Chen et al utilizes a 3D ResNet model to differentiate between true cerebral microbleeds and mimicking structures with high accuracy.[28] Gustafsson et al demonstrates that prostate RT DICOM structures can be accurately labeled on MRI-based sCT with InceptionResNetV2.[80] Finally, Gao et al distinguishes between radiation necrosis and tumor recurrence for gliomas, significantly outperforming experienced neurosurgeons with a CNN.[70]

### 5.2 Treatment Response

The decision to treat with radiation therapy is often definitive. Since radiation dose will unavoidably been delivered to healthy tissue, treatment response and the risk of adverse effects are heavily considered. Further compounding the decision, dose to healthy tissue is cumulative that is complicating any subsequent treatments. In addition, unknown distant metastasis can derail radiation therapy’s curative potential. Therefore, predicting treatment response and adverse effects are of high importance, and significant work has gone into applying deep learning algorithms to prognostic models.

Diffusion weighted imaging (DWI) has attracted strong interest in studies which predict the outcome of radiation therapy. DWI measures the diffusion of water through tissue often yielding high contrast for tumors. Cancers can be differentiated by altering DWI’s sensitivity to diffusion with the b value, in which higher b values correspond to an increased sensitivity to diffusion. By sampling at multiple b-values, the attenuation of the MR signal can be measured locally in the form of apparent diffusion coefficient (ADC) values. A drawback of DWI is that the spatial resolution is often significantly worse than T1W and T2W imaging.[167] Unlike segmentation and synthesis which require highly accurate structural information, high spatial resolution is not necessary for treatment outcome prediction, so the functional information from DWI is most easily exploited in predictive algorithms.

The majority of studies seek to predict treatment outcomes and tumor recurrence. An Xception based model for predicting laryngeal and hypopharyngeal cancer local recurrence with DWI achieves good results in Tomita et al.[230] Zhu et al takes the interesting approach of concatenating DWI histograms across twelve b values to create a “signature image.” A CNN is then applied to the signature image to achieve exceptional performance in predicting pathological complete response.[273] Jing et al, in addition to MRI data includes clinical data like age, gender, and tumor stage to improve predictive performance.[106] Keek et al achieves better results in predicting adverse effects by combining handcrafted radiomics and deep learning features.[119] Other notable papers include Huisman et al which uses an FCN suggesting that radiation therapy accelerates brain aging by 2.78 times[92], Hua et al which predicts distant metastases with an AUC of 0.88,[87] and Jalalifar et al which achieves excellent results by feeding in clinical and deep learning features into an LSTM model[98] An additional study by Jalalifar et al finds the best performance for local treatment response prediction using a hybrid CNN-transformer architecture when compared to other methods. Residual connections and algorithmic hyperparameter selection further improve results.[100]

## 6 REAL-TIME AND 4D MRI

Real-time MRI during treatment has recently been made possible in the clinical setting with the creation of the MRI-LINAC. Popular models include the Viewray MRIdian (ViewRay Inc, Oakwood, OH) and the Elekta Unity (Elekta AB, Stockholm). Electron return effect (ERE), which increasing dose at boundaries with differing proton densities such as the skin at an external magnetic field, guides the architecture of these models.[246] At higher field strengths, the ERE becomes more significant, but MR image quality increases. In addition, a higher field strength can reduce the acquisition time for realtime MRI. Therefore, a balance must be struck. Both the Elekta Unity and Viewray Mridian with 1.5T and 0.35T magnetic fields, respectively, compromise by choosing lower field strengths The Elekta Unity prioritizes image quality and real-time tracking capabilities at the expense of a more severe ERE.[195] The MRI-LINAC has enabled an exciting new era of ART wherein anatomical changes and changes to the tumor volume can be accurately discerned and optimized between treatment fractions. In addition, unique to MRgRT, the position of the tumor can be directly monitored during treatment, potentially leading to improved tumor conformality and improved patient outcomes.[190]

Periodic respiratory and cardiac motion are common sources of organ deformation and should be accounted for optimal dose delivery to the PTV. Tracking these motions is problematic with conventional MRI since scans regularly take approximately 2 minutes per slice leading to a total typical scan time of 20 to 60 minutes.[51] In addition to motion restriction techniques like patient-breath hold, cine MRI accounts for motion in real-time by reducing acquisition times to 15 seconds or less. This is achieved by only sampling one (2D) or more (3D) slices with short repetition times, increasing slice thickness, and undersampling. In addition, the MR signal is sampled radially in k-space to reduce motion artifacts. Capturing a 3D volume across multiple timesteps of periodic motion is known as 4D MRI.[218]

Deep learning methods can further reduce acquisition time by reconstructing intensely undersampled cine MRI slices. In addition to reconstructing from undersampled k-space MRI sequences, several approaches further reduce acquisition time. In the first approach, cine MRI and/or k-space trajectories are used to predict the timestep of a previously taken 4D MRI. However, this method requires a lengthy 4D MRI and does not adapt to changes in the tumor volume over the course of the treatment. Additional approaches include synthesizing a larger volume than cine MRI slice captures to reduce acquisition time, predicting the deformation vector field (DVF) which relays real-time organ deformation information, or determining the 3D iso-probability surfaces of the organ to stochastically determine tumor position if real-time motion adaptation is not possible.

Shown in Table 11, this category is experiencing rapid growth with majority of papers being published within the current year. Notable works include Gulamhussene et al which predicts a 3D volume from 2D cine MRI or a 4D volume from a sequence of 2D cine MR slices. A simple U-Net, introduced in Section 3, is implemented to reduce inference time. The performance degrades for synthesized slices far away from the input slices but achieves an exceptional target registration error.[77] Nie et al instead uses autoregression and the LSTM time series modeling to predict the diaphragm position and to find the matching 4D MRI volume. Autoregression outperforms an LSTM model which could be attributed to a low number of patients.[181] Patient motion is alternatively predicted in Terpestra et al by using undersampled 3D cine MRI to generate the DVF with a CNN with low target registration error.[226] Similarly, Romaguera et al predicts liver deformation using a residual CNN and prior 2D cine MRI. This prediction is then input into a transformer network to predict the next slice.[201] Driever et al simply segments the stomach with U-Net and constructs iso-probability surfaces centered about the center of mass to isolate respiratory motion. These probability distributions can then be implemented in treatment planning.[50]

## 7 OVERVIEW AND FUTURE DIRECTIONS

Innovations in deep learning and MRI are complementary and growing at a fast pace. Shown in Figure 2, the complexity of deep learning algorithms is rapidly increasing. New systems like the MRI-LINAC have allowed for adaptive radiation therapy and real-time MRI during treatment. Despite the successes of the studies reviewed, there are more challenges to overcome.

Many challenges in deep learning applications to MRI are related to limited computational resources. While MRI offers high resolution data which complements deep learning’s big data approach, the typical 3D image size is over one gigabyte of data which means that concessions must be made to apply deep learning methods. These include downsampling the original MRI, forgoing 3D convolution, and processing the images in small patches. As field strengths increase to 7 Tesla and beyond, higher resolution images, as well as generating more powerful and expensive models, computational challenges remain ever-present despite Moore’s Law.[174] Therefore, the task is to most efficiently utilize available computational resources. One source of innovation is the increasing optimization of hardware for computer vision. The improved hardware yields higher performance and efficiency. For example, computer vision tasks have progressed from the central processing unit (CPU) to the graphics processing unit (GPU) and often to the tensor processing unit (TPU). Neuromorphic hardware has demonstrated exceptionally high efficiency and could have applications in real-time MRgRT. Deep spiking neural networks (DSNNs), which are designed for neuromorphic hardware, can more accurately model the human brain than traditional neural networks, allowing for real-time learning and adaptation.[251] For instance, DSNN’s adaptive capabilities could find an application in real-time MRI. Instead of only relying on local context such as in ROI methods and convolution, attention and the transformer allow for direct global context by focusing on relevant regions. Currently, hybrid CNN-transformer architectures are gaining traction by strategically placing transformer layers to improve performance while also keeping the models computationally viable with convolutional layers.[211] In the future, it is foreseeable that pure self-attention models such as the transformer will become state-of-the-art with more powerful hardware and more efficient approaches. This trend towards attention and self-attention models is shown in Figure 2 with a growing interest in attention over the last three years. This could also partially explain the drop in studies using 3D convolution and GAN architectures since more studies are devoting their resources to attention. Another cause for fewer GANs is that nine fewer MRI synthesis studies were written in 2022 in which GANs are the current state-of-the-art method. Finally, diffusion models are an alternative to GANs, which work by gradually adding noise to an image and attempting to recreate it. Although diffusion models are computationally expensive, they can generate more realistic images than GANs and may soon find applications in super-resolution and under-sampled real-time MRI.[44, 139]

Another challenge of deep learning applications to MRgRT is that MRgRT is still a nascent field. For example, prostate brachytherapy often uses fiducial markers designed for CT. Fiducials show exceptional contrast in CT imaging but are difficult to see on MRI and can be challenging for segmentation methods.[215] It is likely with the maturation of the field, designed fiducial markers for MRI will see greater adoption or no longer be necessary for many applications since organ motion can be directly monitored with MRgRT.[124] In addition, high quality public datasets often remain a roadblock. However, the growing number of yearly competitions like the BraTS challenge and public databases like The Cancer Imaging Archive (TCIA) have mitigated this effect.

Deep learning has also enhanced the capabilities of MRI. sCT enables MRI to generate X-ray attenuation information, super-resolution algorithms can reduce the time of acquisition or enhance clinical MRI resolution, and synthetic contrast MRI can achieve similar results to T1C MRI with T1W and T2W sequences. These are actively being researched and should improve with time. The new frontier of MRI research is the MRI-LINAC and 7T MRI. Despite better image details at higher field strengths, the ERE increases in its severity which causes unwanted dose at air-tissue interfaces. Therefore, current MRI-LINAC models operate at 0.35 and 1.5 Tesla while diagnostic MRI is commonly at 1.5 and 3 Tesla. A solution might be to synthesize low tesla MRI to higher field strengths. Similarly, 7T MRI is gaining clinical acceptance for diagnostic imaging. Despite its greater detail, it is not readily available and is more prone to artifacts associated with high strength, non-homogenous magnetic fields making it a good candidates for synthesis algorithms.[227] Synthesis of other modalities from MRI such as ultrasound, positron emission tomography (PET) imaging, and pathological images are also on the horizon as suitable datasets become available.

A prevailing theme is the cross-pollination from different disciplines. The LSTM, GRU, and transformer models were originally developed for language translation and time series estimation but are now common in radiomics, synthesis, and segmentation. The GAN is mainly implemented in synthesis but has found applications in several segmentation architectures. Similarly, super-resolution and CT- and CBCT-based sMRI improve segmentation accuracy. It is foreseeable that MRI-based synthetic functional imaging like PET, single photon emission computed tomography (SPECT), and functional MRI (fMRI) could also improve radiomics performance. Reinforcement learning has found success in two stage segmentation networks by optimally adjusting the bounding box. Monte Carlo Dropout (MCDO) has been implemented in segmentation models to visualize uncertainty but could also be used to visualize synthesis uncertainty.[69] Similarly, it is common to include biometric and MRI scanner manufacturer information in radiomics, and synthesis papers have recently incorporated scanner information to enhance predictions. Clinical data commonly applied to radiomics methods like patient age, prostate specific antigen (PSA) level, and biopsy data may improve MRI synthesis and segmentation methods in the future. In addition, genomics data could refine treatment plans by predicting the radiosensitivity of the patient and tumor, and enhancing the prognostic value of radiomics methods.[113] Another source of inspiration are the sRPSP maps applied in proton therapy which bypass sCT to give accurate attenuation information of protons. It is foreseeable to also synthesize a treatment plan or dose distribution directly from MRI without the need for sCT in an MRI-only workflow. From these developments, inter-field innovation will continue to play an important role in the development of deep learning applications to MRgRT.

## 8 CONCLUSION

In summary, deep learning approaches to MRgRT represent the state-of-the-art in segmentation, synthesis, radiomics, and real-time MRI. These algorithms are expected to continue to improve rapidly and allow for precise, adaptive radiation therapy, and an MRI-only workflow.

## Figures and Tables

**TABLE I.** Brain Segmentation Studies

Study	Year	Target	Network Architecture	Network Features	Imaging Modalities	Patient Number	DSC
**Simon et al[202]**	2022	AVM	U-Net		TOF MRA, T1WC, T2W	23	arteries: 0.86 veins: 0.91 brain: 0.98 CSF: 0.91
**Tian et al[24]**	2022	GBM	U-Net	3D	T1C	20	0.94 ± 0.012
**Bouget et al[72]**	2022	GBM	AGU-Net	Attention	T1W, FLAIR	2134	0.86 ± 0.17
**Momin et al[32]**	2022	Glioma	Retina U-Net	BRATS ROI 3D	T1, T1C, T2W, FLAIR	369	WT: 0.97 ± 0.03 TC: 0.90 ± 0.13 ET: 0.77 ± 0.22
**Ma et al[193]**	2022	Meningioma	CNN	Residual Recurrent Attention	T1C	551	0.89
**Mi et al[163]**	2022	Temporis	U-Net		3.0T T1C	132	0.89
**Huang et al[111]**	2022	Tumor	DeepMedic	3D	T1C	176	0.81
**Chartrand et al[173]**	2022	Tumor	U-Net	3D	T1W	530	2.5–6mm:0.68 ¿ 10mm:0.86
**Yoo et al[19]**	2022	Tumor	U-Net	2.5D	T1C	65	0.75
**Ghaffari et al[130]**	2022	Tumor	Dense U-Net	Dense 3D	T1W, T1C, T2W, T2-FLAIR	15	WT: 0.83 TC: 0.77 ET: 0.60
**Bouget et al[169]**	2021	GBM	nnUNet	BRATS 3D	T1C, T1W	1887	0.87 ± 0.15
**Pan et al[228]**	2021	HC	U-Net	Attention Residual ROI 3D	1.5 T T1W	235	cohort b: 0.76±0.04 cohort c: 0.80±0.015
**Hsu et al[13]**	2021	Tumor	V-Net	Residual 3D	T1C, CECT	511	0.76 ± 0.03
**Shirokikh et al[85]**	2021	Tumor	U-Net	ROI	T1W	1952 images	0.64 ± 0.22
**Lin et al[213]**	2021	Tumor	U-Net	BRATS	T1, T1C, T2W, T2-FLAIR	369	WT: 0.92 ± 0.05 TC: 0.89 ± 0.18 ET: 0.85 ± 0.17
**Huang et al[148]**	2021	Tumor	FCN	BRATS	T2W, T1C, T1, FLAIR	384	WT: 0.86 TC: 0.73 ET: 0.61
**Ahmadi et al [269]**	2021	Tumor	QAIS-DSNN	BRATS	T1, T1C, T2W, T2-FLAIR	145	WT: 0.92 ET: 0.75 TC: 0.80
**Lee et al[89]**	2021	Tumor	Dual Pathway U-Net	3D	1.5 T T1C, T2W	381	0.90±0.05
**Eijgelaar et al[88]**	2020	GBM	DeepMedic	BRATS,3D	T1W, T2W, T1C, FLAIR	751	BRATS: 0.80 Clinical: 0.49 Sparely Labeled:
**Rahmat et al[88]**	2020	GBM	Deep Medic	3D	3.0 T DTI, T2-FLAIR, T1C	80	0.82 ± 0.17
**Ermis et al[53]**	2020	GBM	DenseNet	BRATS Dense	T1W, T1C, T2-FLAIR, T2W	30	WT: 0.83 TC: 0.81 ET: 0.81
**Tang et al[53]**	2020	Glioma	U-Net		CT, T1-FLAIR, T2-FLAIR, T2W, T1C	59	0.818
**Haensch et al[146]**	2020	HC	One Hundred Layers Tiramisu	Dense 2.5D	T1W	45	0.67
**Mlynarski et al[223]**	2020	Multi-Organ	U-Net		T1W	44	hippocampus: 0.88 pituitary: 0.80 brain: 0.99
**Zhou et al[172]**	2020	Tumor	FCN	ROI 2.5D	T1C	934	0.81 ± 0.15
**Bousabarah et al[10]**	2020	Tumor	U-Net		3.0 T T1C, T2W, T2-FLAIR	509	0.60
**Xue et al[250]**	2019	Tumor	FCN		3.0 T T1W	1201	0.85±0.08
**Charron et al[23]**	2018	Tumor	DeepMedic	3D	T1C, T2-FLAIR, T1W	182	0.79
**Liu et al[212]**	2017	Tumor	DeepMedic	BRATS 3D	3.0 T T1C	240	TC: 0.75 ± 0.07 ET: 0.81 ± 0.04

**TABLE II.** HN Segmentation Studies

Study	Year	Target	Network Architecture	Network Features	Imaging Modalities	Patient Number	DSC
Dai et al[40]	2022	Multi-organ	MS R-CNN	Attention Residual ROI 3D	1.5T T1W	60	optic chiasm: 0.61 ± 0.14 oral cavity: 0.92 ± 0.07
Tao et al[147]	2022	NPC	SeqSeg	Attention Residual ROI Recurrent	T1W, T2W, T1C	596	0.80
Deng et al[43]	2022	NPC	DenseNet, V-Net	Dense 3D	3.0T T1W, T2W, T1C (separately)	4478	T1: 0.77±0.07 T2: 0.76±0.07
Zhang et al[200]	2022	NPC	AttR2U-Net	Attention	T1C	93	0.82
Outeiral et al[143]	2022	Oropharyngeal Cancer	U-Net	ROI 3D	T1W, T2W	230	0.64
Jiang et al[104]	2022	Paratoid glands	GAN, U-Net	Attention Residual	3.0T T2W, CT	181	right parotid gland: 0.81 ± 0.05 left parotid gland: 0.82 ± 0.03
Kawahara et al[115]	2022	Paratoid glands, Submandibular glands, lymph nodes	GAN	2.5D	1.5T T2W	55	right lymph node: 0.75 right parotid gland: 0.85
Li et al[199]	2021	NPC	DenseNet	Dense	T1W	30	0.872
Wahid et al[234]	2021	Oropharyngeal Cancer	Residual U-Net	Residual 3D	1.5 T T1W, T2W, DCE, DWI	30	ALL MR Sequences: 0.71 ± 0.12 T1W + T2W: 0.73 ± 0.12
Outeiral et al[198]	2021	Oropharyngeal Cancer	U-Net	3D	T1W, T2W, T1C	171	0.74
Korte et al[127]	2021	Paratoid glands, Submandibular glands, lymph nodes	U-Net	ROI 3D	1.5T T2W	41	LN Lvl IIIL: 0.56 ± 0.10 Left Parotid: 0.86 ± 0.07
Ren et al[79]	2021	Tumors	U-Net		PET, CT, T2W, T1W	153	0.87
Gurney-Champion et al[118]	2020	Lymph nodes	U-Net	3D	1.5 T DWI	48	0.87
Ke et al[147]	2020	NPC	DenseNet	Dense 3D	3.0 T T1W	4100	0.77 ± 0.07
Lin et al[120]	2019	NPC	VoxResNet	Residual 3D	T1W, T2W, T1C, T1-Fat Suppressed	203	0.79

**TABLE III.** Abdomen, Heart, and Lung Segmentation Studies

Study	Year	Target	Network Architecture	Network Features	Imaging Modalities	Patient Number	DSC
Zhang et al[200]	2022	Multi-Organ	U-Net		3T, T2w HASTE	75	DD: 0.88 ± 0.03 Stomach: 0.92 ± 0.02
Ding et al[47]	2022	Multi-Organ	ResU-Net, Active Contour Model	Residual 3D	3T, T2w HASTE	71	DD: 0.49–0.69 Stomach: 0.56–0.77
Luximon et al[107]	2021	Bowel Stomach	Dense U-Net	Dense	.35 T MRI	116	Bowel: 0.90 ± 0.04 Stomach: 0.91 ±0.02
Morris et al[175]	2020	Heart substructures	U-Net	3D	CT, T2W	32	Chambers: 0.88 ± 0.03 Great Vessels: 0.85 ± 0.03 pulmonary veins: 0.77 ± 0.04
Chen et al[120]	2020	Multi-Organ	Dense U-Net	Dense 2.5D	3.0 T T1W VIBE	102	DD: 0.80 ± 0.07 Stomach: 0.92 ± 0.02
Wang et al[237]	2019	Lung Cancer	FCN		3T T2W, prior contours	9	0.82 ± 0.10
Wang et al[236]	2019	Lung Cancer	CNN, GRU	Recurrent Attention	3T T2W	10	Week 4: 0.78 ± 0.22 Week 5: 0.69 ± 0.24 Week 6: 0.69 ± 0.26
Fu et al[120]	2018	Multi-organ	Dense U-Net	Dense 3D	.35 T MRI	120	DD: 0.66 ± 0.09 Stomach: 0.85 ± 0.04

**TABLE IV.** Pelvic Segmentation Studies

Study	Year	Target	Network Architecture	Network Features	Imaging Modalities	Patient Number	DSC
Groendahl et al[75]	2022	Anal Cancer	U-Net	ROI	3.0 T2W, DWI, PET/CT, contrast CT	36	PET, contrast CT: 0.83 ± 0.08 PET, contrast CT, T2W: 0.81 ± 0.08
Shaaer et al[208]	2022	Catheters (Cervix)	U-Net		1.5 T T1W, T2W	20	0.59 ± 0.10
Zabihollahy et al[260]	2022	Cervical Cancer	U-Net	3D	1.5T T2W MRI	123	0.85 ± 0.03
Cao et al[21]	2022	Cervical Cancer	Dual-path CNN	Residual	T2W, CT	65	Small: 0.65 ± 0.03 Medium: 0.79 ± 0.02 Large: 0.75 ± 0.04
Yoganathan et al[255]	2022	Cervical Cancer Multi-Organ	ResNet50, InceptionResNetV2	Residual 2.5D	1.5T T1W	39	GTV: 0.62 ± 0.14
Breto et al[16]	2022	Cervical Cancer Multi-Organ	Mask R-CNN	Residual	0.35T MRIdian	15	GTV: 0.67 ± 0.30
Li et al[144]	2022	Liver, Kidney, Cervical Cancer	nnU-Net		T2W	6	Liver GTV: 0.94 ± 0.01 Kidney GTV: 0.95 ± 0.02 Cervix GTV: 0.97 ± 0.02
Fransson et al[62]	2022	Prostate and OARs	U-Net	ROI	3T, T2W	17	CTV: 0.92 ± 0.03 Bladder: 0.93 ± 0.07 Rectum: 0.84 ± 0.10
Eidex et al[8]	2022	Prostate Cancer	Mask R-CNN	Residual ROI 3D	T1W	77	Prostate: 0.90 ± 0.09 DIL: 0.84 ± 0.12
Li et al[138]	2021	Anal Cancer	U-Net	Attention	Not Specified	304	0.98
Huang et al[90]	2021	Colorectal Cancer	RU-Net	ROI 3D	T2W	64	0.76
Zabihollahy et al[259]	2021	Female Bladder, Rectum, Sigmoid Colon	3D U-Net 3D Dense U-Net	Dense ROI 3D	1.5 T T2W	129, 52	Bladder: 0.94 ± 0.05 Rectum: 0.88 ± 0.04 Sigmoid: 0.80 ± 0.05
Cha et al[21]	2021	Prostate	DeepLabV3 +	Residual	3.0 T T2W, sCT	50	Prostate: 0.89 Bladder: 0.99
Comelli et al[34]	2021	Prostate	E-Net		T1W	85	0.91
Savenije et al[205]	2020	Bladder, Rectum, Femur	Deep Medic	3D	3.0 T T1W	150	Bladder: 0.96 ± 0.02 Rectum: 0.88 ± 0.05 femurs: 0.97 ±0.01
Dai et al[41]	2020	Catheters (prostate)	AGU-Net	Attention 3D	1.5 T T2W	20	Average displacement: 0.37±1.68 mm
Singhrao et al[215]	2020	Fiducials (prostate)	pix2pix (GAN)		T1W	56	0.67
Gustafsson et al[80]	2020	Fiducials (prostate)	HighRes3DNet	Residual 3D	3T T2W	326	0.98 ± 0.002
Sanders et al[203]	2020	Prostate	DenseNet-201		T1W, T2W, T1C	200	Prostate: 0.90 ± 0.04 Bladder: 0.91 ± 0.06 Rectum: 0.96 ± 0.04
da Silva et al[38]	2020	Prostate	Hybrid atlas, active contour		T2W	56	0.85
Chen et al[29]	2020	Prostate cancer	MB-U-Net		3.0 T T2W, ADC, DWI	136	0.63
Zaffino et al[261]	2019	Catheters (Cervix)	U-Net	3D	T2W	50	0.60 ± 0.17
Yang et al[253]	2019	Prostate	MICS-Net		T2W, CT	22	0.83 ± 0.04
Elguindi et al[54]	2019	Prostate and OARs	Deep LabV3+	Residual	T2W	50	CTV: 0.83 ± 0.06 Bladder: 0.93 ± 0.04 Rectum: 0.82 ± 0.05
Nie et al[180]	2019	Prostate and OARS	STRAINet (GAN)	Residual	3.0 T T1W	35	Prostate: 0.91 ± 0.01 Bladder: 0.97 ± 0.01 Rectum: 0.91 ± 0.03
Feng et al[57]	2018	Prostate and OARs	ResNet	Residual	Not specified	40	Prostate: 0.90 ± 0.02 Bladder: 0.96 ± 0.01 Rectum: 0.89 ± 0.03
Wang et al[15]	2018	Rectal Cancer	U-Net	2.5D	3.0T T2W	93	0.74 ± 0.14

**TABLE V.** MRI-based Synthetic CT Studies for Photon Therapy

Study	Year	Site	Network Architecture	Network Features	GAN Type	Imaging Modalities	Patient Number	MAE
**Ranjan et al[196]**	2022	Brain	U-Net		pix2pix	T2W	18	0.03 ± 0.02
**Wang et al[240]**	2022	Brain	U-Net	3D	pix2pix	1.5T T1W	31	MSE: 0.12 ± 0.04 %
**Jabbarpour et al[96]**	2022	Brain	FCN	Residual	cycleGAN	3T T1W, T2W (separately)	189	61.9 ± 22.6 HU
**Scholey et al[206]**	2022	H&N	U-Net	3D	N/A	1.5T T1W	120	whole body: 93.3 ± 27.5 soft tissue: 78.2 ± 27.5 bone: 138.0 ± 43.4 (HU)
**Florkow et al[61]**	2022	Hip Pelvis	U-Net	3D	N/A	3.0 T T1W	30	Femur: 23±24 Pelvis: −15±29 (Mean Error HU)
**Li et al[141]**	2022	Liver	U-Net		N/A	.35T sim	37	35.6 HU
**Lenkowicz et al[135]**	2022	Lung	U-Net		pix2pix	.35T sim	60	54.9 ± 10.5 HU
**Reaunga-mornrat et al[197]**	2022	Pelvis	FCN	Residual	relativistic GAN	High and low res Dixon MRI	45	Median normalized mutual information: 1.28
**O’Connor et al[183]**	2022	Pelvis	U-Net		cGAN	4.0 T T1 VIBE Dixon	40	whole body: 34.7 ± 5.1 bone: 109.4 ± 12.3 soft tissue: 25.2 ± 3.4 (HU)
**Tsekas et al[232]**	2022	Abdomen Torso	U-Net	3D	N/A	1.5 T1W	124	speed: 1.5 seconds per segment
**Zhao et al[267]**	2022	Pelvis	Transformer-CNN hybrid	Residual Transformer	cGAN	T2W	19	45.1 HU
**Hsu et al[86]**	2022	Prostate	U-Net	2.5D	pix2pix	.35T sim	57	pelvis: 30.1 ± 4.2 soft tissue: 19.6 ± 2.3 bone: 158.5 ± 26.0 (HU)
**Olber et al[187]**	2021	Abdomen	Dense U-Net	Dense	GAN	.35T sim	89	No gas: 90 ± 29 HU Gas: 143 ± 29 HU
**Kang et al[114]**	2021	Abdomen Pelvis Thorax	U-Net	Residual 2.5D	cycleGAN	0.35T sim	90	59.2±5.8 HU
**Lerner et al[136]**	2021	Brain	FCN	3D	N/A	Dixon MRI	20	Body: 62.2±4.1 Brain: 9.5±0.7 Bone: 173.8±
**Yuan et al[258]**	2021	Brain	Res U-Net	Residual	N/A	1.5T T1W	30	86.6±34.1 HU
**Liu et al[153]**	2021	Brain	ResNet	Residual	GAN	T1W	12	N/A
**Koerkamp et al[76]**	2021	Breast	Not Specified		revGAN	1.5T T1W	39	106 HU
**Baydoun et al[9]**	2021	Cervix	U-Net		cGAN	T2W	11	115.74 ± 21.84 HU
**Olin et al[189]**	2021	H&N	U-Net	3D	N/A	Dixon MRI	6,17	External: 78 ± 13 HU Local: 76 ± 12 HU
**Liu et al[159]**	2021	H&N	Z-Net, FCN		CycleGAN	Dixon MRI	164	0.04
**Touati et al[231]**	2021	H&N	U-Net		CycleGAN	3.0T T1W	56	45.3 ± 1.9 HU
**Song et al[216]**	2021	NPC	U-Net		N/A	1.5T T1W	35	125.6 HU
**Ma et al[162]**	2021	NPC	U-Net		pix2pix	3.0T T1W	20	102.6 ± 11.4 HU
**Szalkowski et al[220]**	2021	Pelvis	FCN	3D	GAN	T2W	11	72.9 ± 88.1 HU
**Boni et al[17]**	2021	Pelvis	Not Specified		conditional CycleGAN	1.5T, 3.0T T2W	38	59.8 HU
**Bird et al[12]**	2021	Pelvis (ano-rectal)	U-Net		pix2pix	1.5T T2W	90	35.1 ± 7.9 HU
**Yoo et al[256]**	2021	Prostate	FCN	Residual	cycleGAN	T2W	113	96.95 ± 10.32 HU
**Farjam et al[56]**	2021	Prostate	U-Net		N/A	.35T sim	30	whole body: 29.7 ± 4.4, fat: 16.34 ± 2.67 muscle: 23.36 ± 2.85 bone: 105.90 ± 22.80 (HU)
**Cusumano et al[37]**	2020	Abdomen Pelvis	U-Net		pix2pix	.35T sim	120	Abdomen: 78.7 ± 18.5 Pelvis: 54.3 ± 11.9 (HU)
**Liu et al[151]**	2020	Abdomen	U-Net		N/A	Dixon MRI	31	liver: 24.1 spleen: 28.6 lungs: 105.7 vertebral bodies: 110.1 (HU)
**Massa et al[165]**	2020	Brain	Inception V3, U-Net		N/A	1.5T T1W, T2W, T1C, FLAIR (separately)	92	51.2 ± 4.5 HU
**Andres et al[5]**	2020	Brain	HighResNet	Residual 3D	N/A	1.5T, 3.0T T1W, T1C	402	92 ± 23 HU
**Koike et al[126]**	2020	Brain (GMB)	U-Net		pix2pix	T1W, T2W, FLAIR	15	whole body: 108.1 ± 24.0 soft tissue: 38.9 ± 10.7 bone: 366.2 ± 62.0 (HU)
**Olin et al[188]**	2020	H&N	U-Net		N/A	Dixon MRI	11	Body: 94 ± 14 Air: 300 ± 69 Soft tissue: 41 ± 4 bone: 258 ± 51 (HU)
**Largent et al[129]**	2020	H&N	U-Net		GAN	T2W	8	82.8 HU
**Qi et al[194]**	2020	H&N	U-Net		pix2pix	T1W, T2W, T1C, Dixon	45	70.0 ± 12.0 HU
**Klages et al[125]**	2020	H&N	CycleGAN		GAN	mDixon FFE	23	pix2pix: 66.9±7.3 CycleGAN: 82.3±6.4 (HU)
**Tie et al[229]**	2020	H&N (nasopharynx)	ResU-Net	Residual	cGAN	T1W, T1C, T2W	32	75.7 ± 14.6 bone: 194.6 ± 38.9 (HU)
**Bahrami et al[7]**	2020	Pelvis	SegNet (U-Net)	Residual	N/A	3.0T, T2W	15	30.0 ± 10.4 HU
**Florkow et al[60]**	2020	Pelvis	U-Net	3D	N/A	3T T1W	23, 17 dogs	humans: 33 dogs: 35 (HU)
**Kazemirfar et al[116]**	2019	Brain	U-Net		GAN	1.5T T1Gd	77	47.2 ± 11.0 HU
**Lei et al[131]**	2019	Brain Prostate	FCN	Dense	CycleGAN	Brain: T1W Prostate: T2W	44	Brain: 55.7 Prostate: 50.8 (HU)
**Liu et al[150]**	2019	Brain	VGG16	Residual	N/A	1.5T T1W	40	75 ± 23 HU
**Liu et al[156]**	2019	Liver	FCN	Dense	cycleGAN	T1W	21	72.9 ± 18.2 HU
**Olberg et al[186]**	2019	Breast	FCN		GAN	.35T sim	60	16.1 ± 3.5 HU
**Gupta et al[78]**	2019	H&N	U-Net		N/A	3T Dixon	60	81.0 ± 14.6 air: 233.8 ± 28.0 soft tissue: 17.6 ± 3.4 bone: 193.1 ± 38.3 (HU)
**Dinkla et al[48]**	2019	H&N	U-Net		N/A	3T T2W	34	75 ± 9 HU
**Wang et al[244]**	2019	NPC	U-Net		N/A	1.5T T2W	33	whole body: 131 ± 24 soft tissue: 97 ± 13 bone: 357 ± 44 (HU)
**Fu et al[64]**	2019	Pelvis	U-Net	3D	N/A	1.5T T1W	20	2D CNN: 40.5 ± 5.4 3D CNN: 37.6 ± 5.1 (HU)
**Largent et al[128]**	2019	Prostate	U-Net		GAN	3T T2W	39	U-Net: 34.4 ± 7.7 GAN: 34.1 ± 7.5 (HU)
**Emami et al[55]**	2018	Brain	ResNet	Residual	GAN	1.0T T1Gd	15	whole body: 89.3 ± 10.3 tissue: 41.9 ± 8.6 Bone/Air: 240–255 (HU)
**Arabi et al[4]**	2018	Pelvis	U-Net		N/A	3T T2W	39	32.7 ± 7.9 HU
**Chen et al[26]**	2018	Prostate	U-Net		N/A	3T T2W	51	30.0 ± 4.9 HU
**Han[81]**	2017	Brain	U-Net		N/A	1.5T T1W	18	84.8 ± 17.3 HU

**TABLE VI.** MRI-based Synthetic CT Studies for Proton and Boron Therapy

Study	Year	Site	Network Architecture	Network Features	GAN	Imaging Modalities	Patient Number	MAE	Dosimetry
Zimmermann et al[276]	2022	Brain	Res U-Net	Residual	N/A	T1W, T2W, T1C	47	T1: body 79.8 bone 216.3 T2: body 71.1 bone 186.1 T1C: body 82.9 bone 236.4 (HU)	dose parameters within 1%
Zhao et al[268]	2022	Brain	SARU	Attention Residual Boron- therapy	N/A	T1W	104	Head: 67.8 ± 24.3 Skull: 144.0 ± 45.83 Brain: 14.9 ± 21.2 (HU)	2%/2 mm: 0.98 ± 0.01
Wang et al[25]	2022	Brain	Res U-Net	Residual sRPSP	ccGAN (constant cycle)	T1W, T2W, FLAIR	195	42 ± 13 HU	10%/3 mm: 55–60% from chart
Wang et al[238]	2021	Brain	Attention U-Net	Attention	cycleGAN	1.5T, 3.0T T1W	125	65.3±13.9 HU	mean absolute differences: V95 1.1 ± 0.8% 80% beam axis distal falloff 1.1±0.9 mm
Liu et al[152]	2021	H&N	Residual FCN	Residual, Dual Energy CT	label GAN (conditional cycleGAN)	1.5T T1W	57	Low Energy CT: 80.0 ± 18.1 High Energy CT: 80.2 ± 16.3 (HU)	N/A
Maspero et al[164]	2020	Brain	U-Net	2.5D	cGAN	1.5T, 3.0T T1W	60	61 ± 14 HU	2%/2mm: photon 99.5 ± 0.8% proton 99.2 ± 1.1%
Kazemifar et al[117]	2020	Brain, proton	U-Net		GAN	1.5T T1W	77	47.2 ± 11.0 HU	mean absolute difference: CTV <.5% (0.3 Gy) OAR <2% (1.2 Gy)
Florkow et al[59]	2020	Wilms Tumor	U-Net	3D	N/A	1.5T T1W, T2W	54	57 ± 12 HU	2%/2 mm: VMAT >99% PBS (pencil beam scanning) >96%
Liu et al[158]	2019	Liver	FCN	Dense	CycleGAN	T1W	21	72.9 ± 18.2 HU	1%/1 mm: >99%
Shafai-Erfani et al[209]	2019	Brain	FCN	Dense	cycleGAN	1.5 T1W	50	54.6±6.8 HU	2%/ 2 mm, 10% LDT: 98%
Neppi et al[178]	2019	Brain	U-Net	3D	N/A	1.5T T1W	89	137±32 HU	2%/ 2mm: 99.3%
Liu et al[157]	2019	Pelvis	FCN	Dense	cycleGAN	1.5 T2W	17	51.3 ± 16.9 HU	2 mm/2%: 97.95 ± 2.95% mean Bragg peak shift: 0.18 ± 0.07 cm

**TABLE VII.** Synthetic MRI Studies

Study	Year	Site	Network Architecture	Network Features	GAN	Input Modality	Output Modality	Patient Number	Results
Dai et al[39]	2021	H&N	MS-RCNN	Attention Dense ROI 3D	N/A	CT	T1W	108	local DSC 0.77 public DSC: 0.86
Kieselmann et al[266]	2021	H&N	U-Net		CycleGAN	CT	3T T2W	27	DSC: 0.77±0.07
Gotoh et al[73]	2021	Lumbar Spine	U-Net		pix2pix	CT	3T T2W	22	PSNR: 18.4 ± 2.1 MSE: 8876.7 ± 1192.9
Kalantar et al[109]	2021	Pelvis	U-Net		CycleGAN	CT	1.5T T1W	17	PSNR: 18.3 ± 0.2 MAE: 0.057 ± 0.001
Lei et al[133]	2021	Pelvis	FCN	Attention Dense	CycleGAN	CT	T2W	140	DSC: 0.95 ± 0.05
Xu et al[249]	2020	Brain	FCN	Dense 3D	N/A	CT	T1W	391	sMRI MAE: 15.5 sCT MAE: 9.1
Li et al[140]	2020	Brain	U-Net	Attention Dense	N/A	CT	1.5T T1W	34	MAE: 74.2 PSNR: 32.4
Fu et al[67]	2020	Pelvis	U-Net	Attention Dense 3D	CycleGAN	CBCT	T2W	100	bladder 0.96 ± 0.03 prostate 0.91 ± 0.08 rectum: 0.93 ± 0.04 (DSC)
Lei et al[270]	2020	Pelvis	FCN	Attention Dense 3D	CycleGAN	CBCT	T2W	100	bladder 0.95 ± 0.02 prostate 0.86 ± 0.06 rectum 0.91 ± 0.04 (DSC)
Dong et al[14]	2019	Pelvis	U-Net	3D	CycleGAN	CT	T2W	102	Bladder: 0.95±0.03 Prostate: 0.87±0.04 Rectum: 0.89±0.04 (DSC)

**TABLE VIII.** Intramodal MR Synthesis Studies

Study	Year	Site	Network Architecture	Network Features	GAN	Input	Output	Patients	Results
Xie et al[248]	2022	brain (BraTS)	ResUnet	Residual	parallel CycleGANs	1×1×3 mm3 T1W, T2W, T1C, FLAIR (separately)	1×1×1 mm3 T1W, T2W, T1C, FLAIR (separately)	300	SSIM: 0.98 ± 0.01
Xie et al[?]	2022	brain (BraTS)	Retina U-Net	ROI	N/A	T1W	T1C	369	SSIM: 0.99 ± 0.01
Zhou et al[176]	2022	brain	Dual path DenseNet	Dense	GAN	3.0T T2-Flair	4× High Res	237	DSC: .79
Chen et al[27]	2022	pelvis	ResUnet	Residual	CycleGAN	T1W	Rotated T1W	23	Prone MAE: 35.6 ± 4.0 Lateral MAE: 40.5 ± 5.8
Zormpas-Petridis et al[278]	2022	prostate, mesothelioma	ResUnet		N/A	ADC map	ADC Uncertainty Map	44	ADC uncertainty differed by 4.3% for the prostate and 3.7% for mesothelioma
Preetha et al[68]	2021	brain	U-Net		pix2pix	T1W, T2W, FLAIR	Synthetic Contrast	206	median SSIM: 0.82
Chun et al[134]	2019	torso abdomen	FCN	Residual	N/A	.35T MRI		480	SSIM: 0.96

**TABLE IX.** Cancer Detection and Staging Studies

Study	Year	Purpose	Site	Architecture	Network Features	Input Modality	Patient Number	Results
Yang et al[239]	2022	false positive segmentation reduction	Brain	Siamese network, SVM	Residual	T1C	242	AUC: 0.93
Liang et al[145]	2022	NPC Staging	Brain	FCN	Attention Residual	T1C	320	AUC: 0.88
Gustafsson et al[101]	2022	prostate RT DICOM structure classification	Prostate	InceptionResNetV2	Residual	sCT	40	F1: 0.985
Chakrabarty et al[22]	2021	brain tumor classification	Brain	CNN	BRATS 3D	T1C	2105	Internal: 0.85–100 External: 0.73–0.99 (AUC)
Gao et al[70]	2020	Tumor Recurrence or Necrosis	Brain	CNN		T1W, T1C, T2W	146	AUC: 0.96
Zhang et al[264]	2020	suspected lesion classification	Brain	Faster R-CNN, RUSBooster		T1W	121	AUC: 0.79
Zhou et al[271]	2020	brain metastases classification	Brain	CNN		T1W	266	sensitivity: 0.81
Chen et al[28]	2019	cerebrial microbleeds classification	Brain	ResNet	Residual 3D	7T TOF, TOF-SWI MRI	73	AUC: 0.97

**TABLE X.** Treatment Response Studies

Study	Year	Purpose	Site	Network Architecture	Network Features	Input Modality	Patient Number	Results
Huisman et al[92]	2022	Post-radiation brain aging rate	Brain	FCN		3T T1W	32	accelerated aging rate: 2.78 years/year
Keek et al[132]	2022	adverse reaction prediction	Brain	xception, xgboost		T1Gd	1641	AUC: 0.71 recall: 0.80
Jalalifar et al[98]	2022	local tumor control prediction	Brain	InceptionResNet + LSTM + Clinical Feature Fusion	Residual Recurrent	T1Gd, T2-Flair	124	AUC: 0.86
Jalalifar et al[100]	2022	Local metastases treatment response	Brain	Hybrid CNN-Transformer	3D Transformer Residual	T1W, T2-FLAIR	124	AUC: 0.91
Hua et al[87]	2022	Distant Metastases Prediction	H&N	xception		1.5T T1W	441	AUC: 0.88
Tomita et al[230]	2022	laryngeal and hypopharyngeal cancer local recurrence prediction	H&N	xception		1.5T DWI	70	AUC: 0.77
Ottens et al[191]	2022	DCE-MRI hysiological parameter estimation for tracer-kinetic modeling	Pancreas	GRU	Recurrent	3T DCE-MRI	28	random error reduced by factor of 4.8
Zhu et al[49]	2022	rectal cancer treatment response	Rectum	CNN		3T DWI	472	AUC: 0.93
Zhang et al[263]	2021	Distant Metastases Prediction	H&N	ResNet, clinical, regression	Residual	T2W, T1C	189	AUC: 0.80
Jing et al[106]	2021	NPC Risk Score Prediction	H&N	DenseNet + clinical data	Dense 3D	T1W, T2C, clinical data	1846	C-index: 0.67
Jang et al[102]	2021	rectal cancer pathological response	Rectum	ShuffleNet, LSTM	Recurrent	T2W	466	pCR: 0.76 Good Response: 0.72 (AUC)
Jin et al[105]	2021	treatment response	Rectum	CNN	3D	T1W, T2W, T1C, DWI	622	cohort 1: 0.95 cohort 2: 0.92 (AUC)
Gao et al[71]	2021	sarcoma response prediction	Whole Body	VGG-19		.35T DWI	35	accuracy: 0.83
Metz et al[168]	2020	free water correction for Glioblastoma Recurrence prediction	Brain	ANN		DTI	35	AUC: 0.90
Zhang et al[265]	2020	rectal cancer treatment response prediction	Rectum	CNN		DKI, 3.0T T2W	401	pCR: 0.99 treatment response: 0.70 Tumor downstaging: 0.79 (AUC)
Fu et al[65]	2020	rectal cancer treatment response prediction	Rectum	LASSO, VGG19		ADC	43	AUC: 0.73

**TABLE XI.** Real Time and 4D MRI Studies

Study	Year	Site	Purpose	Network Architecture	Network Features	Inputs	Output	Patient Number	Results
Gulamhussene et al[272]	2022	liver	4D	U-Net		2D cine MRI	4D MRI	20	target registration error: 1.2 ± 0.7mm
Xiao et at[247]	2022	liver	4D	U-Net	3D	3D MRI, 4D MRI	High quality 4D MR	39	inference time: 69.3 ± 5.9 ms Anterior-Posterior ROI tracking error: 0.50 ± 0.55
Driever et al[50]	2022	Stomach	Location Probability	U-Net		2D T2W Coronal MRI	3D iso-probability surfaces	18	Median Standard Deviation: organ deformation: 2.0–2.9 mm respiratory deformation: 2.7–8.8 mm
Nie et al[181]	2022	lung	Real Time MRI	auto-regression		2D cine MRI	4D MRI	8	Displacement at 8 Hz: autoregression 0.06 ± 0.02 mm LSTM 0.18 ± 0.06 mm
Shao et al[122]	2022	heart liver	Real Time MRI	FCN		k-space trajectory, prior[122] MRI, undersampled cine MRI	3D MRI	8 cardiac 9 liver	13-spoke k-space cardiac DSC: 0.89 ± 0.02
Tamura et al[222]	2022	lung	Real Time MRI	CycleGAN		2D cine MRI	4DCT	5	3D motion predicted 1.5 seconds in future
Wei et al[245]	2022	liver	Real Time MRI	FCN		T1W Planning MRI, undersampled treatment MRI	Treatment MRI	3	With 12.5% radial undersampling and 15% increase in noise, SNR improved 4.46dB and SSIM by 28%
Frueh et al[33]	2022	Abdomen heart	Real Time MRI	CNN		2D CINE MRI	Local affinity matrices, segmentation	1190 (MRI)	Liver DSC: 0.95/0.96 left ventricle DSC: 0.89/0.90 (forward pass/backwards pass):
Grandinetti et al[74]	2022	liver	Real Time MRI	CNN		Planning Dixon MRI, undersampled MRI	Reconstructed MRI	3	With 12.5% radial undersampling, PSNR: 34.4
Zormpas-Petridis et al[277]	2021	prostate lung	acquisition time reduction	U-Net		subsampled DWI	DWI	39	PSNR: 55.7
Terpstra et al[226]	2021	lung	Real Time MRI	FCN	3D	3D cine MRI	DVF	27	target registration error: 1.87 ± 1.65 mm
Romaguera et al[201]	2020	liver	Real Time MRI	FCN	Residual Recurrent	3T T2W	Next slice	85	vessel position median accuracy: 0.45 mm
Terpstra et al[225]	2020	abdomen	Real Time MRI	FCN		1.5T 2D cine MRI	DVF	135	Standard Reconstruction with undersampling factor of 25: Standard method SSIM: 0.82 ± 0.07 Deep Learning SSIM: 0.80 ± 0.08
Kim et al[123]	2019	torso abdomen	Real Time MRI	FCN	Residual	.35T MRI	Higher spatial, temporal resolution	4	SSIM: 0.89
